# Effect of optimisation to contemporary HFrEF medical therapy with sacubitril/valsartan (Entresto) and dapaglifloziN on left Ventricular reverse remodelling as demonstrated by cardiac magnetic resonance (CMR) Imaging: the ENVI study

**DOI:** 10.1136/openhrt-2024-002933

**Published:** 2024-12-02

**Authors:** Alice Zheng, Robert Adam, Charles Peebles, Stephen Harden, James Shambrook, Ausami Abbas, Katharine Vedwan, Georgina Adam, Paul Haydock, Peter Cowburn, Christopher Young, Jane Long, Michelle Walkden, Simon Smith, Elizabeth Greenwood, Paula Olden, Andrew Flett

**Affiliations:** 1 University Hospital Southampton NHS Foundation Trust, Southampton, UK; 2 Radiology, University Hospital Southampton NHS Foundation Trust, Southampton, UK; 3 Cardiology, University Hospital Southampton, Southampton, UK

**Keywords:** Heart Failure, Heart Failure, Systolic, Magnetic Resonance Imaging, Cardiomyopathies

## Abstract

**ABSTRACT:**

**Introduction:**

Heart failure with reduced ejection fraction (HFrEF) guidelines recommend ‘four pillars’ of medical therapy and device therapy if left ventricular ejection fraction (LVEF) remains ≤35% after 3 months optimum medical therapy.

We conducted the first study to examine the effects of optimisation to contemporary medical therapy on cardiac reverse remodelling, as demonstrated by cardiac magnetic resonance imaging (CMR).

We hypothesised a proportion of patients would undergo beneficial remodelling and LVEF improvement above the threshold for complex device prescription after 6 months.

**Methods:**

HFrEF patients with symptomatic LVEF≤35% despite ACE inhibitor/beta blocker/mineralocorticoid receptor antagonist therapy, and qualified for sacubitril/valsartan switchover were recruited to this single centre prospective study.

CMR was performed at baseline and at follow-up. Clinical, volumetric and outcome data were collected and compared.

**Results:**

Between June 2021 and August 2022, 49 patients were recruited. The majority (80%) were male, mean age 63±14 years. 35 (71%) had non-ischaemic cardiomyopathy. 2 (4%) patients died and 47 were followed up for a median of 7.4 months. There were no heart failure hospitalisations.

Significant reductions were seen in median indexed left atrial volume: 54 mL/m^2^ (41–72) to 39 mL/m^2^ (30–60) (p<0.001); indexed left ventricular end-diastolic volume: 109 mL/m^2^ (74–125) to 76 mL/m^2^ (58–102) (p<0.001); indexed left ventricular end-systolic volume: 74mL/m^2^ (50–92) to 43 mL/m^2^ (27–58) (p<0.001) and mean indexed left ventricular mass: 72±13 g/m^2^ to 62±13 g/m^2^ (p<0.001).

Median LVEF increased by 12 points from 31% to 43% (p<0.001). 29 (59%) patients improved to LVEF>35%. 13 (27%) patients improved to LVEF≥50%.

Median N-terminal pro B type natriuretic peptide (NTproBNP) reduced from 883 ng/L (293–2043) to 429 ng/L (171–1421) (p<0.001).

**Conclusions:**

Optimisation to contemporary HFrEF medical therapy results in beneficial cardiac reverse remodelling and significant improvements in LVEF and NTproBNP at 6 months as demonstrated by CMR. 59% of our cohort no longer met complex device indications. Guidelines suggest re-assessment of LVEF at 3 months, but our data suggests a longer period is required.

**Trial registration number:**

NCT05348226.

**WHAT IS ALREADY KNOWN ON THIS TOPIC**

- Medical therapy for heart failure with reduced ejection fraction (HFrEF) has changed significantly over the last decade and with this, clinical equipoise now exists when interpreting guidelines and deciding the timing of complex device therapy implantation. 2021 ESC HF guidelines recommend re-assessment of left ventricular ejection fraction and decision regarding complex device therapy after 3 months of optimum medical therapy.

**WHAT THIS STUDY ADDS**

- Our study demonstrates the beneficial cardiac reverse remodelling effects of optimisation to contemporary HFrEF therapy, on cardiac MRI for the first time. Our data shows that three out of five patients who meet criteria for complex devices no longer do so after 6 months of optimal medical therapy.

**HOW THIS STUDY MIGHT AFFECT RESEARCH, PRACTICE OR POLICY**

- Our study supports the 2021 American College Cardiology updated consensus pathway that a longer than 3-month period may be required before making complex device prescription in HFrEF patients.

## Introduction

 Since the landmark PARADIGM-HF^1^ and DAPA-HF^2^ trials, heart failure with reduced ejection fraction (HFrEF) guidelines have been updated to incorporate the angiotensin receptor-neprilysin inhibitor (ARNI) sacubitril/valsartan and SGLT2 inhibitors. Contemporary therapy now consists of ‘four pillars’, with ARNI replacing conventional ACE-inhibitor or angiotensin receptor blocker (ARB) therapy where possible, beta blockers, mineralocorticoid receptor antagonists (MRA) and SGLT2-inihibitors. If left ventricular ejection fraction (LVEF) remains ≤35% after 3 months of ‘optimum medical therapy’, and depending on QRS duration, complex device therapy in the form of primary prevention implantable cardioverter-defibrillator (ICD) or cardiac resynchronisation therapy (CRT-D/P) should be considered.^3^

Morbidity and mortality in HFrEF result from complex processes and is unlikely to be predicted by any single parameter, however cardiac and in particular left ventricular, reverse remodelling has been studied as a surrogate marker for efficacy of HFrEF treatments. Favourable features of LV reverse remodelling are associated with reduced mortality risk.^4^

The clinical benefit for conventional HFrEF therapies is well evidenced, however relatively few studies have described associated reverse remodelling processes and relation to outcome. A limited number of historical echocardiographic and radionuclide ventriculography studies showed improvement in LV area and volume parameters with ACE-inhibitors was associated with adverse event reduction. Similarly, decreased LV volumes, mass, improvement in geometry and increased LVEF have been demonstrated with beta-blocker therapy.^5^,^7^

The exact mechanism by which sacubitril/valsartan achieves its profound clinical benefit is not fully understood, however, recent data supports the notion that cardiac reverse remodelling plays a significant role. Echocardiographic studies have shown that switching to sacubitril/valsartan results in LVEF improvement and reduction in left ventricular and atrial volumes, as well as improvement in mitral regurgitation degree and reduction in N-terminal pro B type natriuretic peptide (NTproBNP).^8^,^11^ There are no cardiac magnetic resonance imaging (CMR) studies on the effects of sacubitril/valsartan. Studies on the remodelling effects of SGLT2 inhibitors are also limited, but reduction in left ventricular and atrial volumes, as well as NTproBNP have been reported.^12 13^

At the time of updated heart failure (HF) guidelines recommending ARNI switchover, a level of clinical equipoise existed between HF clinicians regarding the timing of complex device implantation. ‘Three months of optimal medical therapy’ was not clearly defined and open to interpretation, with clinicians considering complex device therapy at different points along the treatment timeline in relation to sacubitril/valsartan initiation and uptitration.

We aimed to conduct the first study to examine the effects of optimisation to contemporary HF medical therapy on cardiac reverse remodelling as evaluated by CMR in HFrEF patients and how this would affect eligibility for complex device prescription.

We hypothesised that optimisation to contemporary HFrEF medical therapy as per updated guidelines would result in beneficial reverse remodelling, improvement in LV volumes and LVEF on CMR imaging. The beneficial reverse remodelling effects at 6 months would result in a proportion of patients no longer meeting criteria for complex device implantation in clinical practice.

## Methods

### Study design and criteria

We conducted a prospective, single centre, single-arm cohort study at a tertiary centre (University Hospital Southampton NHS Foundation Trust, UK) of patients with symptomatic HF and severe left ventricular systolic dysfunction (LVEF≤35%), already established on ‘conventional’ HFrEF therapy.

Inclusion criteria comprised adults ≥18 years with symptomatic (New York Heart Association (NYHA) II–III) HF and LVEF≤35%, despite established treatment with ACE-inhibitor/ARB, beta blocker and MRA who were eligible for, and had been referred for sacubitril/valsartan initiation as per 2016 ESC guidelines.^14^ LVEF≤35% for inclusion was determined by transthoracic echocardiography (TTE).

Exclusion criteria included patients with a pre-existing device, symptomatic hypotension (systolic BP<95 mm Hg), severe renal failure (eGFR<30 mL/min/1.73 m^2^), hyperkalaemia (K>5.4 mmol/L), diagnosis of amyloidosis, sarcoidosis or hypertrophic cardiomyopathy, history of angioedema, myocardial infarction or revascularisation within the last 40 days, valve disease expected to require surgery and life expectancy <2 years.

### Patient and public involvement

The ENVI study concept and design were presented to a patient and public involvement (PPI) group of HF patients already established on sacubitril/valsartan (Entresto). The PPI group unanimously gave positive feedback and had no concerns from a patient perspective. The PPI group also reviewed the Participant Information Sheet and gave written feedback, resulting in an improved final document version.

### Study procedures

All participants had assessment at baseline including clinical and medication history, cardiovascular examination, 12-lead ECG, blood tests, quality of life (QOL) assessments and a cardiomyopathy protocol CMR scan.

Participants were switched to mid-dose (49/51 mg) sacubitril/valsartan (with a 36-hour washout period for those on ACE-I) with a review 2 weeks later to uptitrate to target dose 97/103 mg following clinical review, blood pressure, renal and liver function monitoring. If BP<110 mm Hg, low dose 24/26 mg was chosen to start. Dapagliflozin 10 mg was added following establishment of sacubitril/valsartan if there were no contraindications.

Blood tests included full blood count, renal function and electrolytes (U&Es), liver function tests and NTproBNP. HbA1c was tested prior to dapagliflozin initiation. QOL assessments consisted of Kansas City Cardiomyopathy (KCCQ-12) questionnaire and a 6-minute walk test (6MWT).

**Figure 1:**
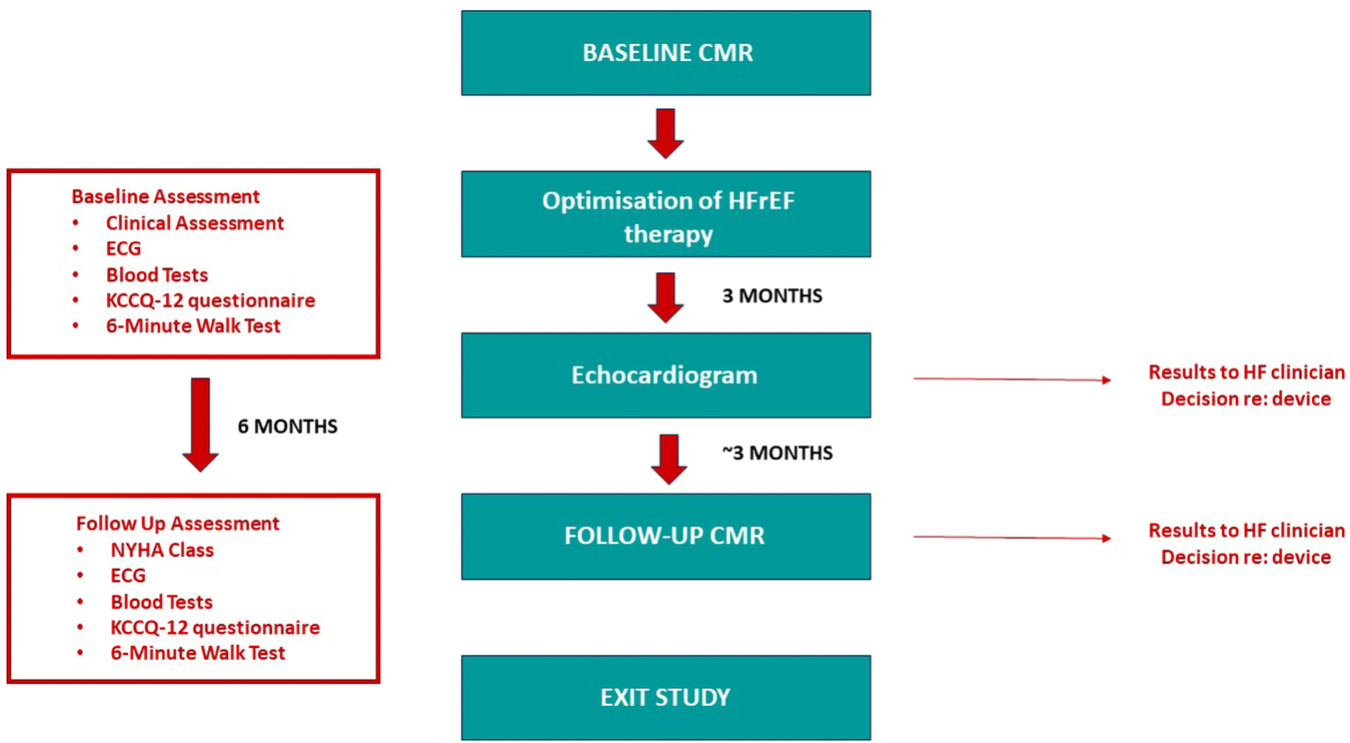
The ENVI study schema. CMR, cardiac magnetic resonance; HF, heart failure; HFrEF, heart failure with reduced ejection fraction; KCCQ-12, Kansas City Cardiomyopathy; NHYA, New York Heart Association.

At 3 months, TTE was repeated to assess LVEF. If LVEF remained ≤35%, the responsible HF clinician decided whether to recommend complex device implantation at that point, reflecting guideline indicated practice. If the participant was added to the device waiting list, repeat CMR was scheduled to occur prior to complex device implantation. Otherwise, follow-up CMR scan was performed 6 months after optimisation to maximum tolerated dose of sacubitril/valsartan±dapagliflozin. Blood tests including NTproBNP, ECG and QOL assessments were repeated at follow-up (figure 1).

Clinical outcome data including death and HF hospitalisation was obtained for the 6-month follow-up period from hospital electronic patient records.

### CMR methods and analysis

CMR scans were performed at baseline and follow-up on 1.5 Tesla Siemens Magnetom Sola scanner with a standardised cardiomyopathy protocol including cines, late gadolinium enhancement, T1 and T2 mapping. All scans were anonymised; baseline and follow-up CMR scans were analysed independently.

All analysis was performed using CVI42 (Circle) software. Volumetric and function data were compared between baseline and follow-up CMR scans.

### Statistical analyses

A sample size of 45 participants was required by power calculation, to achieve 80% power to detect a 5% (SD 8%) change in LVEF, at a significance level of 5%. All statistical tests were performed using IBM SPSS software package V.27.

All variables for each data set were tested for normality using Shapiro-Wilk test, correlated with visual histogram assessment.

For continuous variables, results are reported as mean±SD for normally distributed (parametric) data and median (IQR) for non-parametric data.

Categorical variables are reported as number and percentage frequencies.

Paired t-test was used to compare paired data sets of normally distributed (parametric) continuous variables. Wilcoxon signed-ranks test was used to compare paired data sets of non-parametric continuous variables. A p value of ≤0.05 was considered statistically significant.

## Results

### Study population

A total of 49 patients were recruited between June 2021 and August 2022. The majority, 39 (80%) were male and the mean age was 62.6±13.6 years. Baseline clinical characteristics are shown in table 1.

**Table 1:** Clinical and biochemical characteristics at baseline

Clinical characteristic	n=49
Age (years)	62.6±13.6
Male sex n (%)	39 (80%)
Body mass index (kg/m^2^)	29.5±6.1
Aetiology	
Non-ischaemic cardiomyopathy n (%)	35 (71%)
Ischaemic cardiomyopathy n (%)	14 (29%)
Functional classification	
NYHA Class II n (%)	35 (71%)
NYHA Class III n (%)	14 (29%)
Comorbidities	
Hypertension n (%)	23 (47%)
Ischaemic heart disease n (%)	21 (43%)
Type 2 diabetes n (%)	14 (29%)
History of atrial fibrillation n (%)	20 (41%)
Chronic kidney disease n (%)	9 (18%)
Peripheral vascular disease n (%)	2 (4%)
Cerebrovascular disease n (%)	2 (4%)
Chronic obstructive pulmonary disease n (%)	3 (6%)
Physiology	
Mean systolic blood pressure (mm Hg)	129±16
Mean diastolic blood pressure (mm Hg)	76±11
Mean heart rate (bpm)	75±12
Sinus rhythm—baseline ECG	34 (69%)
Left bundle branch block—baseline ECG n (%)	5 (10%)
Right bundle branch block—baseline ECG n (%)	3 (6%)
Laboratory	
Mean haemoglobin (g/L)	140±14
Median urea (umol/L)	8.3 (6.3–9.4)
Mean creatinine (umol/L)	96±25
Mean potassium (mmol/L)	4.3±0.4
Median NTproBNP (ng/L)	883 (293–2043)
HFrEF medical therapy	
ACE-inhibitor/angiotensin receptor blocker n (%)	49 (100%)
Beta blockers n (%)	47 (96%)
Mineralocorticoid receptor antagonist (MRA) n (%)	48 (98%)
SGLT2 inhibitor n (%)	28 (57%)

Most patients, 35 (71%) had a diagnosis of non-ischaemic cardiomyopathy (NICM) and 14 (29%) had a diagnosis of ischaemic cardiomyopathy.

Among clinical characteristics, 23 (47%) had hypertension, 14 (29%) had Ttype 2 Ddiabetes, and 20 (41%) had Aatrial Ffibrillation. At baseline, 35 patients (71%) were NYHA Class II.

HFrEFheart failure with reduced ejection fractionNTproBNPN-terminal pro B type natriuretic peptideNYHANew York Heart Association

All patients were already receiving optimised conventional HFrEF therapy at time of recruitment, with 49 (100%) on ACE-I/ARB, 47 (96%) on beta blocker and 48 (98%) on MRA therapy at baseline. 28 patients (57%) were already on an SGLT2 inhibitor (dapagliflozin).

At baseline, the median LVEF as measured by CMR was 31% (21–35) and the median NTproBNP was 883 ng/L (293–2043).

### Follow-up and clinical outcomes

At follow-up, all patients had been optimised to maximally tolerated dose of sacubitril/valsartan and 38 (78%) were on dapagliflozin.

Two (4%) patients died (OOH cardiac arrest) during the study period. Both died within 40 days of recruitment; one of whom had previously been offered a CRT-D but declined. The remaining 47 patients were followed up for a median period of 7.4 (6.7–7.9) months. There were no HF hospitalisations during the study period.

Median NTproBNP reduced from 883 ng/L (293–2043) to 429 ng/L (171–1421) (p<0.001).

#### Cardiac reverse remodelling outcomes

At 3 months, 44 patients attended for TTE. 26 (59%) of these patients still had an LVEF of ≤35% at this point. At 6 months, 29 (59%) patients had demonstrated LVEF improvement on follow-up CMR scan to >35%.

There was no apparent difference between remodelling observed by aetiology: 21 (60%) of the 35 patients with non-ischaemic cardiomyopathy and 8 (57%) of the 14 patients with ischaemic cardiomyopathy demonstrated LVEF improvement to >35%. Of the 39 male patients, 22 (56%) remodelled; of the 10 female, 7 (70%) remodelled. The sample size was however too small to perform formal subgroup analysis.

12 (24%) patients who had severe LVEF≤35% on TTE at 3 months went on to remodel further to an improved LVEF>35% on follow-up CMR. 13 (27%) had an LVEF>50% at follow-up.

**Figure 2:**
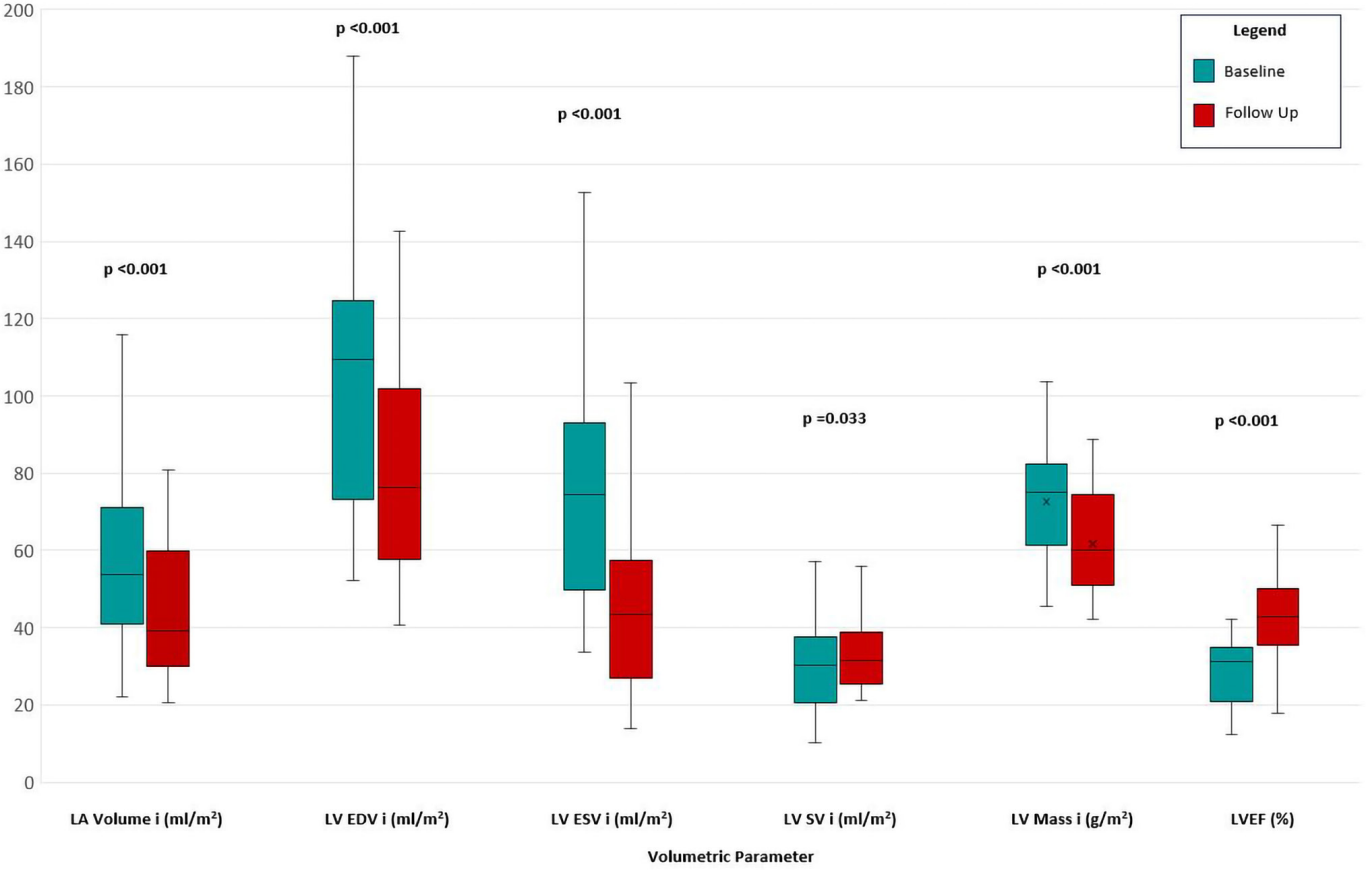
Comparison of indexed volumetric parameters and LVEF between baseline and 6-month follow-up cardiac magnetic resonance scans following optimisation to contemporary heart failure with reduced ejection fraction medical therapy. LA, left atrial; LV, left ventricular; LVEDVi, indexed left ventricular end diastolic volume; LVEF, left ventricular ejection fraction; LVESVi, indexed left ventricular end systolic volume; LVSVi, indexed left ventricular stroke volume.

Table 2 shows all volumetric results at baseline and follow-up CMR scans, indexed to body surface area. Figure 2 demonstrates the change between follow-up and baseline results.

**Table 2:** Comparison of left ventricular and left atrial cardiac magnetic resonance parameters reported as mean±SD or median (IQR) between baseline and follow-up scans

	Baseline (n=49)	Follow-up (n=47)	P value
Left ventricle			
LVEDVi (mL/m^2^)	109 (74–125)	76 (58–102)	<0.001
LVESVi (mL/m^2^)	74 (50–92)	43 (27–58)	<0.001
LVSVi (mL/m^2^)	30 (21–38)	32 (25–39)	0.033
LVEF (%)	31 (21–35)	43 (26–50)	<0.001
LV Mass i (g/m^2^)	72±13	62±13	<0.001
Septal thickness (mm)	9 (8–11)	10 (8–12)	0.307
Left atrium			
LA volume i (mL/m^2^)	54 (41–72)	39 (30–60)	<0.001
LA diameter (mm)	43±9	40±8	0.001

LVEDViindexed left ventricular end diastolic volumeLVEFleft ventricular ejection fractionLVESViindexed left ventricular end systolic volumeLV Mass iindexed left ventricular massLVSViindexed left ventricular stroke volume

At follow-up, there were significant reductions in median indexed left ventricular end diastolic volume (LVEDVi) from 109 mL/m^2^ (74–125) to 76 mL/m^2^ (58–102) (p<0.001) and median indexed left ventricular end systolic volume (LVESVi) from 74 mL/m^2^ (50–92) to 43 mL/m^2^ (27–58) (p<0.001).

The median indexed left ventricular stroke volume (LVSVi) increased from 30 mL/m^2^ (21–38) to 32 mL/m^2^ (25–39) (p=0.033) and the median LVEF increased from 31% (21–35) to 43% (26–50) (p<0.001).

The median indexed left atrial volume (LAVi) reduced from 54 mL/m^2^ (41–72) to 39 mL/m^2^ (30–60) (p<0.001) and the mean indexed left ventricular mass (LV Mass i) reduced from 72±13 g/m^2^ to 62±13 g/m^2^ (p<0.001).

**Figure 3:**
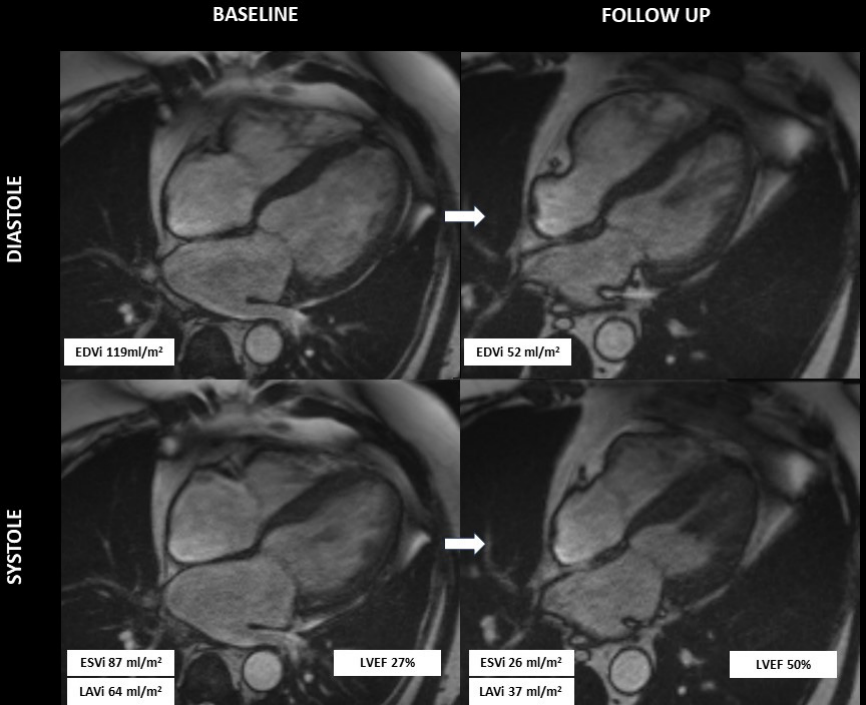
Cardiac magnetic resonance 4-chamber cine still captures in diastole and systole demonstrating beneficial left ventricular and left atrial reverse remodelling after 6 months of optimisation to contemporary heart failure with reduced ejection fraction therapy. LA, left atrial; LAVi, indexed left atrial volume; LV, left ventricular; EDVi, indexed end diastolic volume; LVEF, left ventricular ejection fraction; ESVi, indexed end systolic volume.

Figure 3 shows an example of CMR 4-chamber cine still captures in diastole and systole, before and after optimisation of HFrEF therapy.

#### LVEF improvement and complex device therapy

44 patients attended for TTE at 3 months, of which 26 had a reported LVEF≤35%. After individual assessments by their responsible HF clinician, six patients were offered a complex device (ICD or CRT-D/P) at that point.

One patient had CRT-D implanted within the month, and had their follow-up CMR brought forwards. Four patients were listed for a device, but due to standard NHS waiting times, their allocated device implant date occurred after their follow-up CMR.

At the end of the study period, a total of nine (18%) patients received a complex device, as they had not positively remodelled on their follow-up CMR. After shared decision making processes, four patients declined a device and three were still undecided.

At baseline, eight patients had a bundle branch block on ECG (three with RBBB, five with LBBB). At follow-up, four patients no longer met criteria for cardiac resynchronisation therapy as their LVEF had improved, and one patient no longer had LBBB. No patients developed a new bundle branch block over the study period. The median QRS duration decreased from 110 ms (104–125) at baseline to 106 ms (98–119) at follow-up (p=0.01).

Over half of our cohort, 29 (59%) had demonstrated beneficial reverse remodelling after optimisation to contemporary HFrEF therapy, with improved LVEF>35% on follow-up CMR, whereby a complex device was no longer indicated.

### QOL outcomes

A total of 40 (85%) patients completed 6MWTs both at baseline and at follow-up (some unable to complete due mobility/pain). There was significant improvement in mean 6MWT distance walked (361±133 m vs 393±115 m; p=0.03) for the patients who had improved LVEF to >35% after 6 months of optimised HFrEF therapy. There was no difference in the patient group who still had LVEF≤35% (323±69 m vs 321±94 m; p=0.96).

The comparison of QOL assessments between baseline and follow-up are shown in table 3.

**Table 3:** Comparison of quality of life parameters at baseline and follow-up

6-minute walk test (6MWT)			
	Baseline (m)	Follow-up (m)	P value
All patients (n=40)	348±117	370±113	0.09
LVEF>35% at FU (n=27)	361±133	393±115	0.03
LVEF≤35% at FU (n=13)	323±69	321±94	0.96

Kansas City Cardiomyopathy Questionnaire (KCCQ-12)			
	Baseline score	Follow-up score	P value
All patients (n=47)	75 (46–90)	78 (50–94)	0.07
LVEF>35% at FU (n=29)	73 (44–92)	78 (43–96)	0.09
LV≤35% at FU (n=18)	75 (56–81)	78 (52–90)	0.42

6-minute walk test (6MWT) and Kansas City Cardiomyopathy Questionnaire (KCCQ-12) scores are shown for all patients; patients who had improved LVEF>35% at follow-up, and those with LVEF remaining ≤35% at follow-up.

FUfollow-upLVEFleft ventricular ejection fraction

At baseline, 35 (71%) of patients were NYHA Class II and 14 (29%) were NYHA III. After 6 months, 13 (28%) of patients reported being asymptomatic (NYHA I) at follow-up assessment. The proportion of patients reporting both NYHA II and III symptoms reduced to 53% and 19%, respectively.

## Discussion

After 6 months of optimised contemporary HFrEF medical therapy, we found significant beneficial left ventricular and left atrial reverse remodelling, resulting in an improvement in median LVEF of 12 points.

Adverse cardiac remodelling is intrinsic to the progression of HFrEF. Compensatory mechanisms triggered by myocardial injury and stress result in molecular, cellular and interstitial changes of the myocardium. As left ventricular dysfunction progresses, cardiac dimensions, volumes and mass increase and the heart’s geometry changes from an elliptical to spherical shape, causing secondary mitral regurgitation, exacerbating preload and further dilatation.^15^

### Sacubitril/valsartan and reverse remodelling

Potential beneficial effects of sacubitril/valsartan on cardiac reverse remodelling have thus far all been assessed by echocardiography.

EVALUATE-HF found a reduction from baseline in the sacubitril/valsartan group of LVEDVi, LVESVi and LAVi, as well as improved diastolic function, compared with enalapril observed at 12 weeks. They did not however, demonstrate a difference in LVEF.^16^

PRIME found, in patients with left ventricular dysfunction and chronic secondary mitral regurgitation, a significant decrease in effective regurgitant orifice area and regurgitant volume, as well as reduction in LVEDVi in the sacubitril/valsartan group at 12 months, compared with valsartan.^17^

PROVE-HF found that magnitude and speed of reduction of NTproBNP concentration correlated with improvement of cardiac volume and function at 12 months. They reported a mean increase of 9 points in LVEF at 12 months from 28% to 37%; as well as improvements in LVEDVi, LVESVi, LAVi, and E/E′ ratio.^8^

A study that focused on association of reverse remodelling of patients treated with sacubitril/valsartan found that those who demonstrated LV reverse remodelling (LVEF>45% or LVESV volume reduction by >15%) had a significantly improved prognosis compared with those who did not respond.^18^

### SGLT2 inhibitors and reverse remodelling

There is limited but emerging data on the effects of SGLT2 inhibitors on cardiac remodelling. A meta-analysis of nine SGLT2 inhibitor trials in HF reported reductions in absolute LV volumes (LVEDV, LVESV) and indexed LV mass, and an increase in mean LVEF of +2% (p=0.003), particularly in HFrEF patients. The majority of trials included were empagliflozin and canagliflozin. The only included trial evaluating dapagliflozin (REFORM) found no significant changes in cardiac remodelling parameters on CMR in patients with diabetes and HFrEF.^13 19^ The DAPA-MODA study which included patients with HF regardless of LVEF treated with dapagliflozin and evaluated cardiac remodelling parameters with echocardiography, found global reductions in indexed left ventricular volumes, mass and indexed left atrial volume at 180 days. There was also significant reduction in NTproBNP at 180 days.^12^

### Reverse remodelling and prognosis

Left ventricular ejection is a powerful predictor of cardiovascular outcomes, with a recognised inverse relationship between LVEF and mortality. A meta-analysis to quantitively assess the relationship between therapy-induced changes in LV remodelling and longer-term outcomes included 30 mortality trials and 88 remodelling trials of HFrEF therapies. It found a significant association between short-term trial-level therapeutic effects on LV remodelling, that is, increase in mean LVEF and reductions in mean LVEDV and LVESV, and beneficial longer-term trial level effects on mortality.^4^

### Contemporary HFrEF therapy effects on reverse remodelling and function on CMR

Our study is the first to report the effects of optimisation to contemporary HFrEF therapy on cardiac reverse remodelling in a cohort of symptomatic patients who had not demonstrated improvement despite established conventional therapy, using CMR.

SGLT2 inhibitors became included in international HF guidelines during the recruitment period and so our study reflects real world practice. 57% of our cohort were already on dapagliflozin at baseline, which increased to 78% at follow-up. The predominant intervention to optimise HFrEF therapy was the switchover to maximum tolerated dose of sacubitril/valsartan, in all patients.

Previous reverse remodelling studies as discussed earlier, have used echocardiography to evaluate cardiac volumes, function and response to therapy. CMR has now emerged as the gold standard for accurately assessing LV volumes, mass, function and cardiac anatomy with excellent reproducibility, compared with other imaging modalities. Unlike echocardiography, LVEF with CMR is calculated by true volumetric evaluation, without any geometric assumption of the ventricle, resulting in higher reproducibility and accuracy, when assessing response to therapy.^20 21^

We have found a 30% decrease in median LVEDVi and a 42% decrease in median LVESVi; as well as an absolute increase of 12 points in median LVEF after 6 months. Our data complements previously published echocardiographic studies and shows beneficial improvement in LV geometry and improvement in function as demonstrated by CMR, a key mechanism in achieving the prognostic benefits in these HFrEF patients.

### Left atrial volume and left ventricular mass

Other putative mechanisms for improved prognostic outcomes include reductions in indexed left atrial volumes and left ventricular mass.

Left atrial enlargement and dysfunction are established markers of both systolic and diastolic dysfunction and predictors of poor cardiovascular outcome including stroke, atrial fibrillation, HF and mortality.^22^

Increased left ventricular mass is an independent risk factor for adverse cardiovascular events including myocardial infarction, stroke, HF hospitalisation and death.^23 24^

In our study, we have found a 28% reduction in median LAVi and a 14% reduction in mean indexed LV mass following 6 months of optimised HFrEF medical therapy.

### Reverse remodelling and complex devices

Current ESC guidelines recommend consideration of complex device implantation (ICD or CRT-P/D) for patients with symptomatic HFrEF and LVEF≤35% after at least 3 months of optimum medical therapy, to prevent sudden cardiac death or to optimise dyssynchrony in the case of CRT.^3^

At baseline, all patients in our study had LVEF≤35% despite establishment on conventional HF therapy of ACE-I/beta blocker/MRA and thus were all complex device candidates.

At 3 months after optimisation, 47% had improved LVEF>35% on echocardiography. By 6 months, 59% had an LVEF>35% on follow-up CMR and no longer qualified for a complex device. This included one patient who no longer had an LBBB on ECG.

The PROVE-HF investigators also found that among the cohort of patients who met eligibility for an ICD at baseline, 32% had improved their LVEF to >35% by 6 months and 62% to >35% by 12 months, after initiation of sacubitril/valsartan. The risk of sudden cardiac death was 2% within 6 months for PARAGIM-HF and <1% at 1 year for PROVE-HF.^25^

Analysis into the modes of death in PARADIGM-HF showed that 44.8% (n=561) of deaths were classed as ‘sudden death’ and a 20% reduction in risk was observed in the sacubitril/valsartan group, compared with enalapril (HR 0.80; 95% CI 0.68 to 0.94; p=0.008).^26^ An anti-arrhythmic effect has been described in two prospective studies of HFrEF patents with ICD/CRT-Ds in situ and remote monitoring. Following switchover from ACE-I/ARB to sacubitril/valsartan, episodes of non-sustained ventricular tachycardia, sustained ventricular tachycardia and appropriate ICD shocks were significantly decreased.^27 28^

In 2021, the American College Cardiology (ACC) published an updated consensus stating that LV re-assessment to determine device decision should occur between 3 and 6 months following optimisation of medical therapy, with a shorter period for those at ‘higher risk, for example, LVEF<30%, evidence of ventricular ectopy or ischaemic cardiomyopathy’ and 6 months for those at ‘lower risk’.^29^

Our data which includes patients all established on sacubitril/valsartan and most on dapagliflozin, found an even higher proportion improving LVEF to >35% at 6 months. It suggests that a longer period before re-assessment such as 6 months, would mean a significant number of HFrEF patients may avoid the need, and the associated complications of complex device implantation. This could also mean significant cost reduction to the healthcare system.

It is however, not known whether transitioning from an LVEF≤35% to >35% means that a complex device would not benefit a patient, either in terms of sudden cardiac death risk reduction, or reduction in HF symptoms in the case of CRT. The current LVEF cut-off values for recommendation of complex devices are derived from the inclusion criteria of landmark device trials. More recently, DANISH demonstrated no overall benefit in all-cause mortality of primary prevention ICDs in non-ischaemic cardiomyopathy with LVEF≤35%. Conversely, the presence of mid-wall LGE has been shown to be associated with a ninefold increase in sudden cardiac death/aborted sudden cardiac death in patients with non-ischaemic cardiomyopathy and an LVEF>40%.^30^

There is growing evidence that using LVEF cut-off as the sole arbiter of ICD recommendation is insufficient. The aetiology of cardiomyopathy and other factors for example, genetics, LGE and family history are likely to play significant roles. Further research to guide a more multiparametric risk assessment of patients with cardiomyopathy is required moving forward, however this discussion is beyond the scope of this paper.^31^

### Limitations

The main limitations of our study were the relatively small sample size and the single-arm nature, without control. Given the proven prognostic benefits of ARNI and SGLT2 Inhibitors in this population, a control arm would not have been ethically possible.

The majority (80%) of our study population was male. This is similar to the proportion of males in both PARADIGM-HF (78%)^1^ and DAPA-HF (77%).^2^ Women are unfortunately consistently underrepresented in clinical trials, and this is a recognised limitation when interpreting our conclusions.^32^

### Future directions

Further studies with a longer follow-up period would be interesting, to assess whether the improvements seen in LVEF are sustained over time and whether positive remodelling is also associated with a reduction in risk of adverse outcomes. A larger cohort size would allow for studying potential differences in cardiac reverse remodelling responses to HFrEF therapy between non-ischaemic and Ischaemic aetiologies.

## Conclusion

After 6 months of optimisation to contemporary medical therapy for HFrEF patients, to include maximum tolerated dose of sacubitril/valsartan and dapagliflozin, there were significant improvements to both left ventricular and left atrial remodelling parameters as demonstrated by CMR imaging in both non-ischaemic and ischaemic cardiomyopathy patients.

There were reductions in indexed left ventricular volumes and mass, indexed left atrial volume and a significant increase of median left ventricular ejection by 12 points. There was also a significant reduction in median NTproBNP concentration.

59% of this cohort, all of whom had LVEF≤35% at baseline, demonstrated beneficial left ventricular reverse remodelling by 6 months, and no longer met criteria for complex device implantation.

## Data Availability

Data are available upon reasonable request.
